# Behavioral and Electrophysiological Responses Evoked by Chronic Infrared Neural Stimulation of the Cochlea

**DOI:** 10.1371/journal.pone.0058189

**Published:** 2013-03-07

**Authors:** Agnella Izzo Matic, Alan M. Robinson, Hunter K. Young, Ben Badofsky, Suhrud M. Rajguru, Stuart Stock, Claus-Peter Richter

**Affiliations:** 1 Department of Otolaryngology, Feinberg School of Medicine, Northwestern University, Chicago, Illinois, United States of America; 2 Department of Biomedical Engineering, University of Miami, Miami, Florida, United States of America; 3 Department of Otolaryngology, University of Miami, Miami, Florida, United States of America; 4 Department of Molecular Pharmacology and Biological Chemistry, Northwestern University, Feinberg School of Medicine, Chicago, Illinois, United States of America; 5 Department of Biomedical Engineering, Northwestern University, Evanston, Illinois, United States of America; 6 The Hugh Knowles Center, Department of Communication Sciences and Disorders, Northwestern University, Evanston, Illinois, United States of America; University of South Florida, United States of America

## Abstract

Infrared neural stimulation (INS) has been proposed as a novel method for neural stimulation. In order for INS to translate to clinical use, which would involve the use of implanted devices over years or decades, the efficacy and safety of chronic INS needs to be determined. We examined a population of cats that were chronically implanted with an optical fiber to stimulate the cochlea with infrared radiation, the first known chronic application of INS. Through behavioral responses, the cats demonstrate that stimulation occurs and a perceptual event results. Long-term stimulation did not result in a change in the electrophysiological responses, either optically-evoked or acoustically-evoked. Spiral ganglion neuron counts and post implantation tissue growth, which was localized at the optical fiber, were similar in chronically stimulated and sham implanted cochleae. Results from chronic INS experiments in the cat cochlea support future work toward INS-based neuroprostheses for humans.

## Introduction

The goal for neuroprostheses is to restore neural function to a condition having the fidelity of a healthy system. An emerging technology for neural stimulation is the application of pulsed infrared radiation to stimulate small populations of neurons [1], [2] in vivo in a non-contact manner [3], [4]. Infrared neural stimulation (INS) relies on optical absorption by water, which results in a transient temperature rise. Wavelengths used for INS are typically 1840–1890 nm and 2120 nm. These wavelengths are available from diode and solid state (Ho:YAG) lasers and have moderate water absorption coefficients, ranging from approximately 10 cm^−1^ to 50 cm^−1^ [5]–[8]. Pulse durations are selected such that INS operates in thermal confinement, where the laser energy is deposited and the temperature increase occurs before heat dissipates from the tissue [9], [10]. INS can have better spatial resolution of stimulation as compared to conventional electrical stimulation [3], [11]–[13]. An added benefit of INS is the typical lack of electrical stimulation artifact, which can challenge simultaneous electrical stimulation and recording. Recent in vitro studies have shown that INS reversibly alters the electrical capacitance of the plasma membrane by local heating and depolarizing the target cell [14].

One field where INS has been extensively studied is the stimulation of cochlear neurons, as an alternative to electrical stimulation used in cochlear implants (CI) [15]–[18]. It has been suggested that cochlear implant users’ music appreciation and speech recognition in noisy listening environments would benefit from an increased number of independent perceptual channels [19]–[21]. Research has been directed at increasing the number of independent CI channels by more discretely stimulating cochlear spiral ganglion neurons [22].

Several strategies have been employed to increase the spatial selectivity of electrical stimulation (ES) over a conventional monopolar stimulation paradigm. For instance, multipolar electrode configurations and nerve-penetrating electrodes can increase the spatial selectivity of stimulation [22]–[25]. Each of these methods has drawbacks, such as marginal user improvement for multipolar stimulation in cochlear implants [26] and inflammation and impaired neural conduction with penetrating electrodes in motor neurons [27], [28]. It has been shown that the spatial selectivity of cochlear INS is similar or better when compared to an acoustic tone [11], [13], [29]. The spatial resolution of INS is especially appealing for neuroprostheses as INS likely provides more independent channels for simultaneous stimulation of a neural system than is currently available electrically.

To date, studies involving INS have focused solely on acute stimulation periods, lasting up to 10 hours [16], [30]. In order for INS to translate to clinical use, which would involve the use of implanted devices over years, the efficacy and safety of chronic INS needs to be determined. An additional question that remains for an INS-based neuroprosthesis is whether INS induces a perceptual event. Studies on INS in sensory systems have used electrophysiology and immunohistochemistry as markers for neural depolarization [15], [29], [31], but these data provide no evidence for a functionally relevant event or perception. Here, we present behavioral, electrophysiological, histological, and imaging results from chronic implantation and chronic stimulation studies in cats.

## Methods

### Ethics Statement

Care and use of animals was conducted in accordance with guidelines in the NIH Guide for the Care and Use of Laboratory Animals. The study was approved by the Animal Care and Use Committee of Northwestern University (Protocol Number: 2011-1334). All surgery was performed under anesthesia and all efforts were made to minimize pain.

### Animal Model

Adult, normal hearing cats (Class A, 4–6 months old, Liberty Research, Inc.), which were purpose-bred for research, were used for the experiments. Cochlear function was tested with the acoustically-evoked auditory brainstem response (aABR). Normal hearing animals were used, rather than chemically or genetically deaf animals, to assess the physiological state of cochlea and any damage that resulted from implantation and chronic stimulation.

### Pre-implantation Auditory Test

Each cat scheduled for a cochlear implantation underwent an auditory test to document pre-implantation thresholds. The cat was sedated with Telazol**®** (5–10 mg/kg, intramuscular) and supplemented with atropine (0.04 mg/kg, subcutaneous). Then, the cat was placed in a soundproof booth and given supplemental heat to maintain core body temperature. Cochlear function was screened using auditory brainstem responses (ABRs) to acoustic tonebursts (aABR). Three needle electrodes were placed under the skin to obtain ABRs by subtracting ipsilateral mastoid from vertex potentials measured relative to a ground electrode placed in the neck. The contralateral ear was blocked during testing to reduce any possible cross-talk. Acoustic stimuli were generated by a voltage command presented at a rate of 4 Hz to a Beyer DT 770-Pro headphone, which was calibrated with a Bruel and Kaer 1/8-in. microphone. The speculum of the speaker was placed directly in front of the ear canal (quasi free field). The tonebursts started at 32 kHz and were decreased in 2 steps/octave over 5 octaves. Sound levels, at each frequency, began at the loudest speaker output and were decreased in 5 dB steps. The loudest speaker output varied from 71 dB to 101 dB, depending on the frequency. The ABR electrodes were connected to a differential amplifier (ISO-80, WPI) with a high-input impedance (>10^12^ Ω), set at 10,000x amplification. Further amplification (10x) and filtering (0.3 to 3 kHz) of the signal was performed by a digital filter (IP90, Frequency Devices). The sampling rate was 200 kHz and 100 trials were averaged. Threshold was defined as an ABR that was visible above the noise floor of the recordings, which was ∼0.5 µV. At the conclusion of the hearing test, the animal woke up and returned to its home cage.

### Cochlear Implantation

The animal was premedicated with Telazol**®** (2–4 mg/kg, intramuscular), butorphanol (0.4 mg/kg, subcutaneous), and atropine (0.04 mg/kg, subcutaneous). An intravenous catheter was placed (22G) in the cephalic vein and IV fluids, supplemented with 2.5% dextrose, were given throughout the length of the procedure. Anesthesia was maintained with isoflurane (1%–3%). The cat was placed into a head holder and the surgical area was aseptically prepared. A “C” shaped incision was made behind the left pinna and the bulla was surgically accessed. An opening, approximately 5 mm×5 mm, was drilled in the bulla to visualize the basal turn of the cochlea. The cochleostomy was drilled using a 0.5 mm-diameter drill bit attached to a motorized drill (MicroTorque II, WPI). A custom-made optical fiber probe, consisting of one flat-polished 200-µm-diameter optical fiber (FIP200220240, Polymicro) inserted through implantable grade plastic tubing (0.04″ ID, 0.07″ OD, S-54-HL, Tygon), was inserted through the bulla and oriented towards the cochleostomy. Only the distal tip of the optical fiber, which protruded past the end of the tubing approximately 2–3 mm, was introduced into the cochlea to within 200–1600 µm of the modiolar wall. The optical fiber was oriented towards the spiral ganglion cells. The optical fiber placement was confirmed posthumously with micro-computed tomography (microCT, see below for details).

A small incision, ∼1 cm long, was made in the skin between the scapulae. The proximal end of the optical fiber probe was tunneled under the skin from the bulla to the scapular incision, where the probe exited. The incisions were closed in several layers with an interrupted suture. Post-operatively, the animal was monitored daily and received buprenex (0.005–0.01 mg/kg, subcutaneous, 2x/day for 2–3 days) and meloxicam (0.1 mg/kg, oral, 1x/day for 3–4 days). No vestibular deficits were seen in any of the animals.

### Post-implantation Auditory and Laser Test

Approximately 2 weeks after surgery, the animal was tested for hearing thresholds and laser responses. aABR responses were recorded as described above with the same acoustic stimuli (see “Pre-operative auditory test” for details on sedation and recording procedures). Statistical analysis was completed on the aABR data to determine any significant elevation of threshold following implantation. A Student’s t-test was used to determine statistical significance, with the null hypothesis indicating no threshold difference between the two conditions. A one-tailed test was used for the post-operative measurement since a threshold decrease following cochlear implantation was highly unlikely.

After the auditory thresholds were established, the implanted optical fiber was connected to an external bench-top laser (Capella R-1850, Lockheed Martin Aculight). The laser was operated at 1855 nm wavelength, 100 µs pulse duration, and 10 Hz repetition rate. Energy output ranged from 5–89 µJ/pulse, as measured at the tip of the optical fiber in air (using a Coherent J-50-LP-1A energy sensor). Optically-evoked ABRs (oABRs) were recorded for different energies. At the conclusion of the post-operative test, the animal woke up and returned to its home cage. The laser repetition rate for the oABR measurements was selected at 10 Hz for two reasons. First, a large body of cochlear INS data was recorded at 10 Hz stimulation rate, which can be compared to the current experiments. Additionally, higher INS stimulation rates evoke smaller amplitude potentials, making quantification more difficult.

### Chronic Laser Stimulation

Following the post-operative testing, by at least one day, the implanted optical fiber was connected to a miniaturized laser and battery-powered stimulator (Lockheed Martin Aculight) that had a center wavelength of 1850 nm, and operated at 100 µs pulse duration, 200 Hz repetition rate, and 12 µJ/pulse energy (measured at the tip of the optical fiber in air using a Coherent J-50-LP-1A). At these laser settings, the battery life of the stimulator was ∼8 hours. The animal wore a jacket with a pocket, which contained the laser and stimulator. The implanted optical fiber exited the percutaneous scapular incision and was fed through a hole in the dorsal aspect of the jacket to connect to the laser.

The animal was awake and active in its home environment during the entire time of chronic stimulation. At the time the miniaturized laser was turned on, video footage of the animal was recorded to document any behavioral responses to the stimulation. Chronic stimulation then commenced for 4–8 hours/day for up to 30 days.

### Behavioral Observation

For the videotaping sessions, one cat at a time was released from its cage and allowed into the room. Video footage was acquired with a hand-held digital video camera. Taping sessions were completed at the onset of laser stimulation, with each session lasting from 2–5 minutes. Video of each cat was recorded prior to and immediately following the activation of the laser stimulator. After taping was complete, the video was transferred to a computer (MacBook Pro) and Quicktime software was used to view the video. The video was analyzed for head turns and ambulation. An event was defined as a deviation of the head or upper torso of more than 30° from the midline position. Comparisons were made between the implanted (left) and non-implanted (right) side, and between the pre- and post-laser activation period. A paired Student’s t-test was used to test statistical significance. A second observer, blinded to the experimental conditions, also analyzed the video footage for head turns and ambulation.

### Terminal Experiment

At the end of the chronic stimulation period, a final aABR was recorded to document any threshold changes. Statistical analysis was completed on the aABR data to determine any significant elevation of threshold following chronic stimulation. A Student’s t-test was used to determine statistical significance, with the null hypothesis indicating no threshold difference between the post-implantation aABR and the post-stimulation aABR. A two-tailed test was used since either a threshold increase or decrease could have occurred following chronic stimulation. Seven of the 10 cats used in this study had complete aABR recordings (pre-implantation, post-implantation, post-stimulation) on which to perform data analysis.

The animal was then used in a terminal experiment on the non-implanted cochlea (data not shown here). At the conclusion of the experiment, the animal was euthanized with Euthasol**®** (1.0 mL, intravenous) and the tissue was subsequently fixed by intra-cardiac perfusion of 0.9% saline solution, followed by paraformaldehyde (4% in phosphate buffer, pH 7.4). The tissue was then postfixed for at least 12 hours at 4°C in either 4% paraformaldehyde or in a mixture of 2.5% glutaraldehyde and 1.5% paraformaldehyde, in phosphate buffer, pH 7.4 [32]. The chronically implanted cochlea was removed from the temporal bone, with the implanted optical fiber in place, and fixed in paraformaldehyde solution for at least 24 hours. Following tissue fixation, the cochlea was then imaged using microCT and was embedded and sectioned for histology.

### MicroCT Imaging

The placement of the optical fiber was determined using the MicroCT-40 system (Scanco Medical). For scanning, the fixed cochlea was washed in Ringer’s lactate and subsequently placed in a plastic tube. The cochlea was imaged with the microCT system, operating at 45 kV tube voltage, 88 µA tube current, and 300 ms integration time for each projection. A complete description of the scanner can be found elsewhere (Scanco Medical Web site, www.scanco.ch). Each of the 120–180 slices was imaged with 500 projections of 1,024 samples. The 30.7-mm-diameter field of view was reconstructed with a 1,024×1,024 grid; the slices were contiguous and approximately 30 µm thick. The reconstructed slices were imported into ImageJ and were converted into a stack of TIFF files.

### Histology

Following fixation and microCT imaging, the cochlea was decalcified using one of two methods. The first method was a 10% EDTA bath (pH 7.0) over several months, with weekly changes of the EDTA solution, followed by 1–2 weeks in a solution of 0.4 M formic acid and 0.08 M sodium citrate. The second method was incubation at 38°C in 0.1 M hydrochloric acid and 1 M formic acid for 24–28 hr. The decalcified cochlea was dehydrated in graded ethanol baths from 50–100%, in steps of 10%. Baths were repeated three times for 30 min at each ethanol concentration. Tissue was cleared with at least three changes of xylene until the tissue was fully translucent. The tissue was infiltrated with paraffin wax embedding medium (Paraplast Xtra, Leica Biosystems) under vacuum at 56°C, with four baths of 15 min each. Cochleae were oriented in a tissue mold and embedded in the paraffin wax. Tissue blocks were exhaustively sectioned at 10 µm-thick slices with a rotary microtome. Tissue sections were collected on Superfrost Plus (VWR) glass slides and adhered by overnight incubation at 58°C. For staining, sections were de-waxed and re-hydrated through baths of xylene (3 changes, 2 min each), 100% ethanol (2 changes, 2 min each), 95% ethanol (3 changes, 2 min each) and water. Sections were stained with hematoxylin or toluidine blue, the reverse of the de-waxing regimen was performed, and coverslips applied with Permount (Fisher Scientific). Digital photomicrographs were taken using standard transmitted light microscopy.

Cochlear histological sections were evaluated for the presence of the basilar membrane, tectorial membrane, outer hair cells, inner hair cells, and supporting cells. Additionally, tissue growth in the cochlea was quantified. Spiral ganglion neurons were counted and the density of neurons in Rosenthal’s canal, opposite to the optical fiber, was determined [33], [34]. The first section that contained spiral ganglion neurons at the basal cochlear end was displayed in Adobe Photoshop. An additional layer was inserted on the image and a circle of the approximate size of the neuronal cell nuclei marked the layer for each cell counted. Characteristic landmarks were also added to align the image on the next section. After the cells in section 1 were counted, the corresponding image of section 2 was opened. The inserted layer from section 1 was then copied onto section 2. The images were aligned so that the landmarks superimpose. This clearly identified the cells from section 1 that were already counted. Another layer was inserted on the image, and the cells in section 2 that were not counted in section 1 were then marked on the inserted layer. This then identified the already counted cells in section 2 and allowed them to be omitted in the counts of section 3, and so on. In this way, every neuron in the cochlea could be counted without systematic errors due to over- or under-counting. To simplify the counting, we did not distinguish between type I and type II spiral ganglion neurons. The cross sectional area of Rosenthal’s canal was measured on digital images using ImageJ (Wayne Rasband, NIH). Area measurements of Rosenthal’s canal were obtained by tracing the shortest line encircling the spiral ganglion neurons. The total number of pixels within a circumscribed area was calculated and converted into square millimeters. Neuron density was calculated by dividing the neuron counts by the corresponding area. The corresponding volume density was calculated by dividing the number of neurons counted by the volume (sum of the areas times the slice thickness).

## Results

Adult, normal hearing cats (4–6 months old) were used for the experiments. A total of 10 cats of either sex were used for the study: 3 were used for chronic implantation and stimulation, 3 were implanted such that the optical fiber was non-stimulating but laser pulses were delivered to the cochlear fluid, and 4 served as sham implantations with a dummy optical fiber assembly. Cochlear function was tested with acoustically-evoked auditory brainstem responses (aABRs). Normal hearing animals were used, rather than chemically or genetically deaf animals, to assess the physiological state of cochlea and any damage that resulted from implantation and chronic stimulation.

### Behavioral Observation

**Figure 1:**
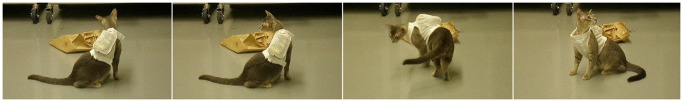
Behavioral responses to cochlear INS seen in the chronically implanted cat. Still images acquired after the chronically implanted laser stimulator was turned on show the cat exhibiting a behavioral response. The cat was implanted in the left cochlea. Three cats exhibited head turns toward the left and ambulation in a circle towards the left. Analysis of the video footage prior to the onset of laser stimulation did not reveal any behavioral patterns or systematic preference towards one side. The panel represents video frames taken 30–42 s after the laser was activated. The laser operated at 1850 nm, 200 Hz repetition rate, 100 µs pulse duration, and 12 µJ/pulse (maximum possible). Full video footage can be seen in supplemental information online (Movie S1 and S2).

**Figure 2:**
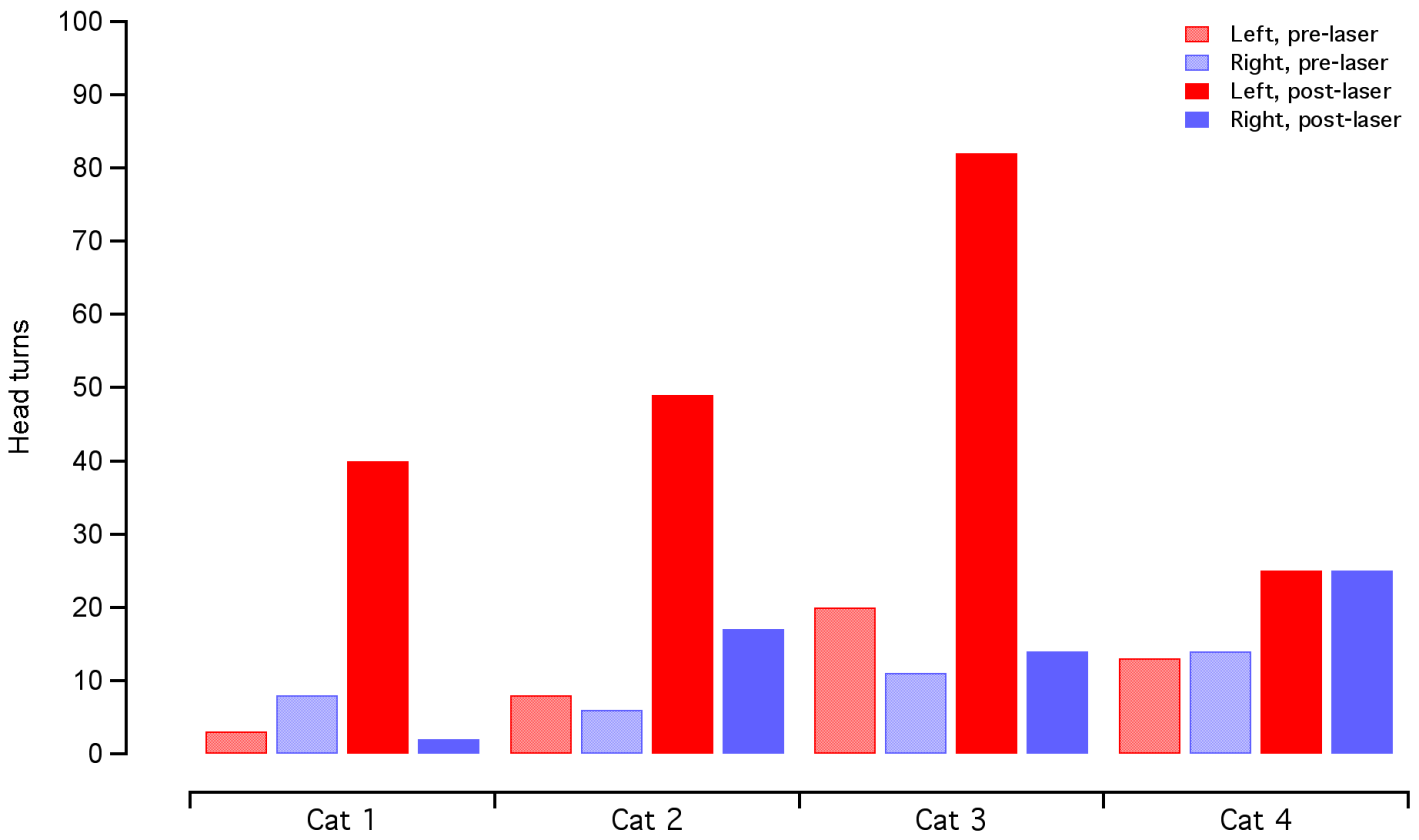
Behavioral analysis show an increase in turns toward the implanted side after laser activation. In three animals, there is a drastic increase in head turns toward the left (implanted) side following the laser activation. The total time of head turns shows some variability between animals for the same ear and same time period. In one animal, cat 4, there was no evidence of a behavioral response, as measured by head turns. The counts for head turns are the same for the right and left side post-laser activation. Red bars indicate head turns toward the left (implanted) side and blue bars indicate head turns toward the right. Shaded bars represent the 2 minutes before laser activation and solid bars represent the 2 minutes after laser activation.

**Figure 3:**
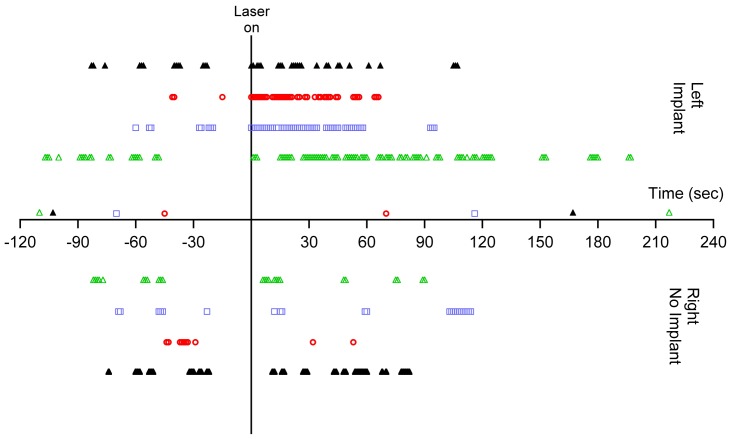
Timing of the behavioral response correlates with laser activation. Raster plots of behavioral responses in the implanted cats show the timing of head turns. Colored symbols show the cats in which a behavioral response was evident (same data as presented in cats 1–3 in figure 2). These cats also had measurable oABRs (see next figure). The black symbols are an example of a cat that did not show a behavioral response or an oABR (cat 4 in figure 2). The data at negative time points show the occurrence of events before the laser activation. None of the cats show a preference of movement towards the right side (below the x-axis) or left side (above the x-axis). After the laser activation at t = 0 s, the colored symbols show a clear increase in frequency of head turns, compared to the pre-laser condition, with most of the movement towards the implanted (left) side. The frequency of the movements decreases 1–2 minutes after laser activation. In the control cat, represented by the black symbols, there is no systematic preference for head turns before or after the laser is activated. The data markers just above the x-axis indicate when video footage began and ended for each cat.

After surgical implantation of an optical fiber in the cochlea, three cats showed behavioral changes indicating that the animal had an auditory perception in response to the laser. The animals turned their head in the direction of the implanted (left) side and ambulated towards the implanted side (Fig. 1; Movie S1 and S2). The laser operated at 1850 nm, 200 Hz repetition rate, 100 µs pulse duration, and 12 µJ/pulse (maximum possible from miniaturized laser). No systematic behavioral patterns or ambulation preferences were seen prior to the onset of laser stimulation. Raster plots of the head turns, as a function of time pre- and post-laser activation, are shown in Fig. 2. A paired Student’s t-test showed no difference in number of head/body turns toward each side (left/right, p = 0.67, n = 3) during the pre-laser period. Following laser activation, there was an increase in the number of head turns between 300–1200% of the pre-activation period (Fig. 2). The head turns were seen for ∼1–2 minutes following the laser activation and decreased in frequency with increasing laser-on time (Fig. 3). A paired t-test indicated that the number of head turns toward the implanted (left) side after laser onset was significantly different than the number of turns towards the non-implanted side (p = 0.05) and the number of turns toward the implanted side before laser onset (p = 0.03). Analysis by a blinded observer confirmed these findings. At 5 minutes post-activation, the animals had adapted their behavior to the laser stimulation. None of the behaving cats showed vestibular side effects, which include excessive salivation and persistent head tilt. Ambulation in a circle did not appear to be a vestibular deficit because the gait was steady.

### Auditory Brainstem Responses (ABRs)

**Figure 4:**
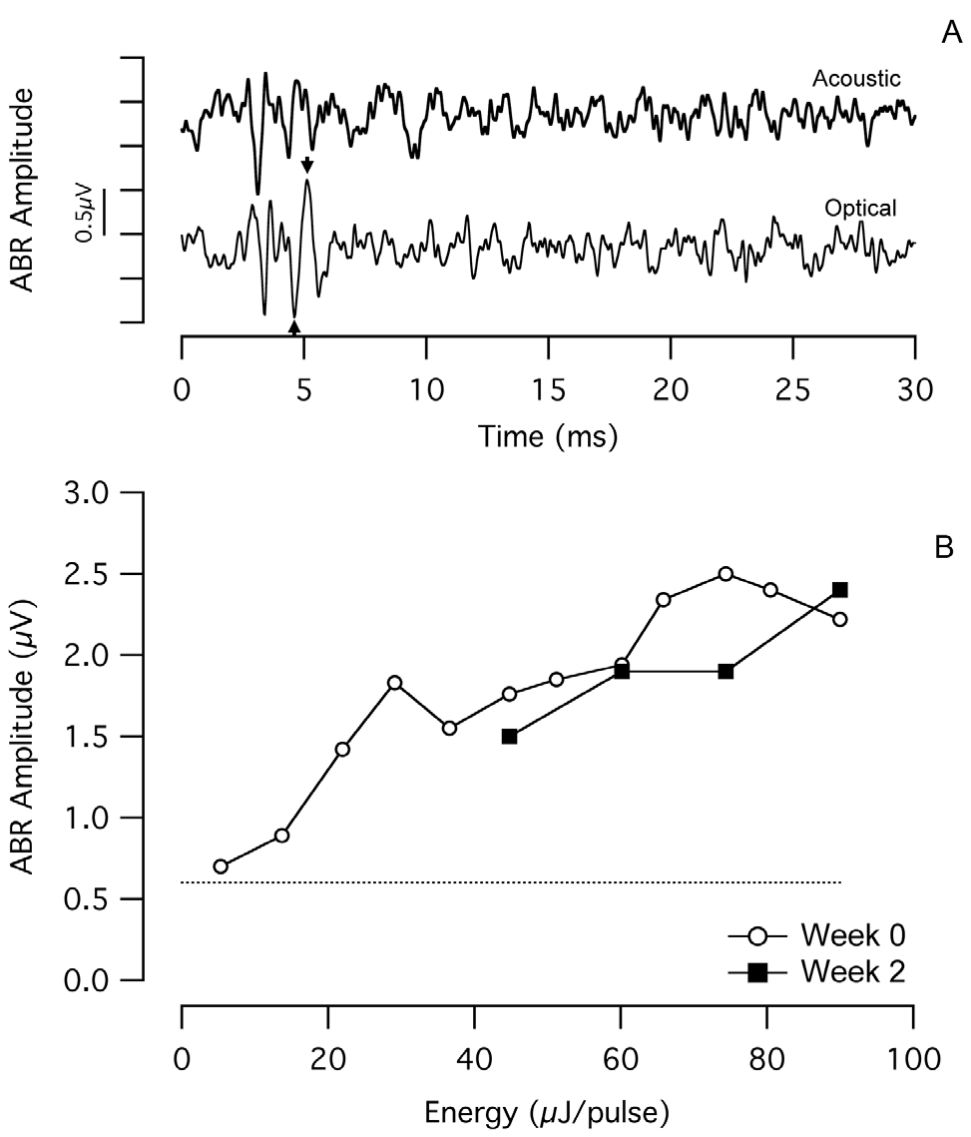
Constant oABR amplitude over several weeks of chronic INS. (a) An optically-evoked auditory brainstem response (oABR) evoked by the chronically implanted optical fiber, with an amplitude of ∼1.6 µV in response to 36 µJ/pulse laser stimulation (optical fiber coupled to the bench-top Capella laser). The arrows indicate how the oABR amplitude was calculated. For comparison purposes, an acoustically-evoked ABR (aABR) recorded in the same session is presented. The acoustic stimulus was an 8 kHz tone pip at 100 dB SPL. The onset of the laser pulse and the onset of the acoustic toneburst occur at t = 0 ms. Each trace reflects the average of 100 sequential responses. (b) The dose-response curves show a monotonic increase in oABR amplitude with increasing laser energy. After 2 weeks of chronic stimulation, oABR amplitudes are similar to those measured at the onset of chronic stimulation. Low energy measurements could not be acquired at week 2 due to significant EMG contamination of the recordings. The dotted line shows the typical noise level of the measurement system. The laser operated at 1855 nm, 10 Hz repetition rate, and 100 µs pulse duration.

ABRs were recorded while stimulating the cochlea optically (oABR) and acoustically (aABR). Optical stimuli were used to monitor long-term efficacy of INS. Acoustical stimuli were used to monitor overall cochlear function. Detectable oABRs were only recorded from the cats that demonstrated a behavioral response to optical stimulation (n = 3). Fig. 4a shows a representative oABR recorded from a chronically stimulated cat. Fig. 4b shows the dose-response curve recorded from the same cat. In the example shown, the radiant energy for the oABR threshold was ∼5 µJ/pulse, and thereafter the evoked amplitude increased monotonically. When using a bench-top laser to evoke oABRs (operating at 1855 nm wavelength, 100 µs pulse duration, and 10 Hz repetition rate), energies up to 75 µJ/pulse were tested. The open circles indicate the energy versus oABR amplitude at the onset of the chronic laser stimulation and the filled squares show the corresponding function that was recorded after 2 weeks of chronic stimulation. The oABR amplitudes were very similar from the 0 week measurement to the 2 week measurement. At the 2-week time point, oABRs could not be measured at lower laser energies due an unusually large amplitude noise in the recordings.

**Figure 5:**
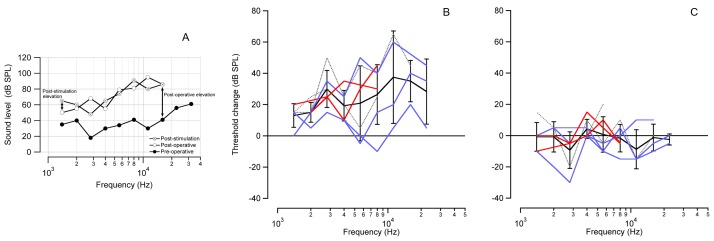
Acoustic thresholds assess the physiological state of the cochlea. Acoustic thresholds were assessed with aABR recordings pre- and post-operatively, as well as at the conclusion of the stimulation period. (a) The graph shows representative data acquired from an individual cat, exhibiting an elevation of the aABR threshold 2 weeks after implantation, especially at high frequencies near the site of the cochloeostomy. No further elevation of the aABR threshold is seen after 1 month of laser stimulation. Arrows indicate the calculation of the post-operative elevation (post-operative aABR – pre-operative aABR) and the post-stimulation elevation (post-stimulation aABR – post-operative aABR). (b) The average of all cats shows a post-operative aABR threshold elevation, which increases with increasing acoustic frequency. Note, the cochleostomy was drilled at ∼20 kHz location in the cat cochlea. (c) There is no significant post-stimulation elevation of the aABR across all stimulated animals. Seven of the 10 cats used in this study had complete aABR recordings (pre-implantation, post-implantation, post-stimulation) on which to perform data analysis. For (b) and (c): The red lines represent the stimulated cats that had the optical fiber pointed towards the spiral ganglion neurons, the blue lines represent cats that received INS pulses directed away from the spiral ganglion neurons, and the gray dotted lines show the sham implanted cats. Some individual lines do not span the entire range of tested frequencies due to missing data. This was usually caused by an elevation of the auditory threshold beyond the output range of the speaker. The average ± standard deviation is shown in black. The zero line on the y-axis in shows the condition of no change in ABR threshold.

Cochlear function was monitored in the chronically implanted cat population using the non-invasive acoustically-evoked auditory brainstem response (aABR). aABR data show that the implantation caused an increase in acoustic threshold, especially at high acoustic frequencies. An example is shown in figure 5a. This finding is not unexpected as the location of the cochleostomy is in the basal turn of the cat cochlea, which encodes high acoustic frequencies. Threshold shifts were quantified for implanted cats (stimulated, non-stimulated, and sham) across 5 octaves. The “post-operative elevation” measured the difference between the post-operative aABR and the pre-operative aABR. The “post-stimulation elevation” measured the difference between the post-stimulation aABR and the post-operative aABR (a diagram of the measurements is shown in Fig. 5a).

The average aABR threshold for all cats showed a post-operative elevation. The changes were larger for higher acoustic frequencies (Fig. 5b), with a maximum elevation of ∼35 decibels sound pressure level (dB SPL) above 11 kHz. All frequencies examined from 1.4 kHz to 22.4 kHz showed a statistically significant post-operative elevation of the aABR threshold (Student’s one-tailed t-test; *H_0_* = 0; *p*≤0.04). At 32 kHz, almost all cats (6/7) did not have a measurable aABR during the post-operative test. The average post-stimulation aABR thresholds showed no overall shift in ABR threshold following 1 month of laser stimulation (Fig. 5c). None of the frequencies measured had average threshold shift that was statistically different from zero (Student’s two-tailed t-test; *p*≥0.08). This shows that the chronic laser stimulation did not have a significant effect on the auditory thresholds of the implanted cochleae.

### microCT Imaging

**Figure 6:**
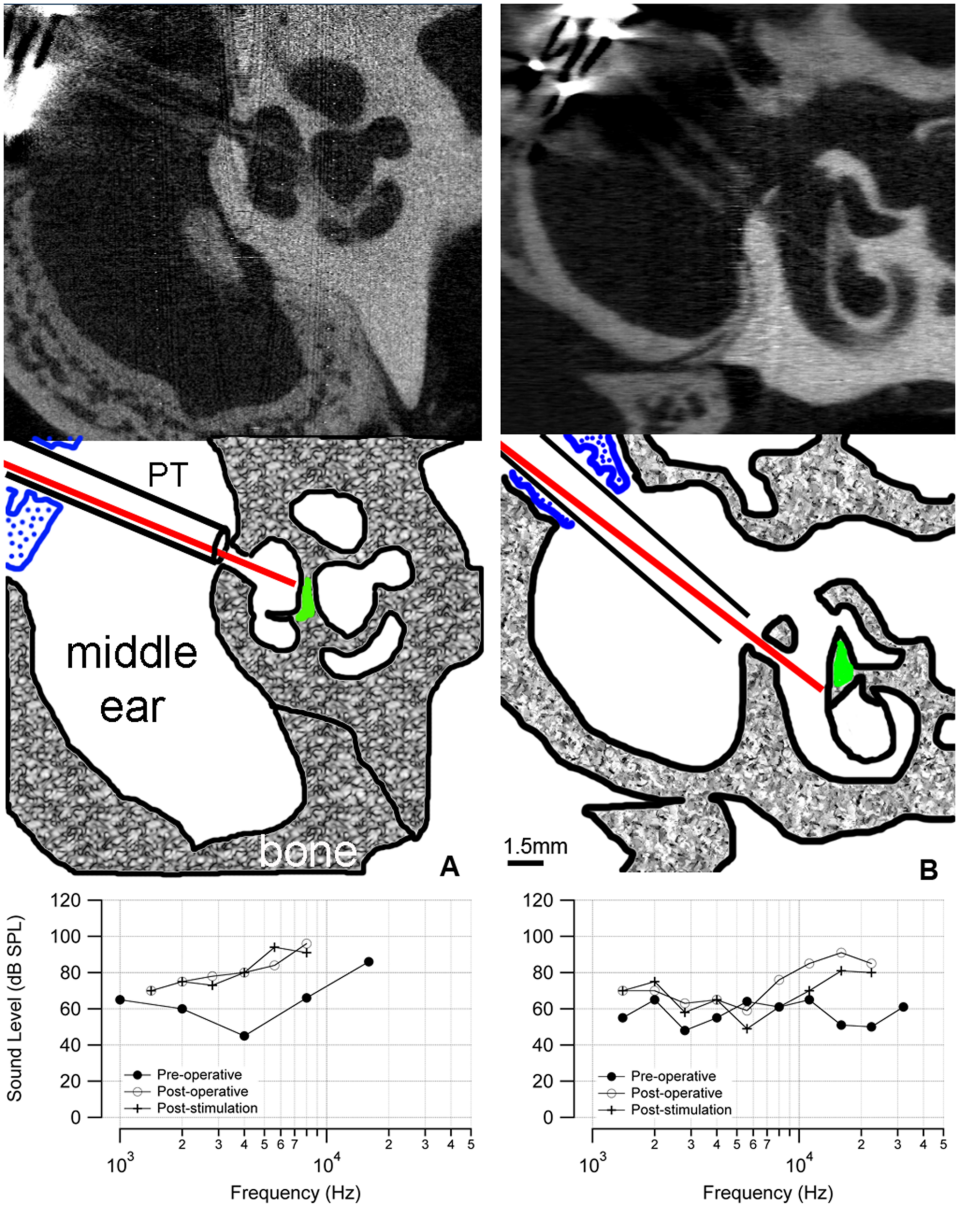
microCT images of chronically implanted cat cochleae. (a) A microCT image of a stimulated cat cochlea shows the placement of the optical fiber. The diagram (middle) indicates the optical fiber (red) passing through the cochleostomy that has been drilled in the bony cochlear wall. The optical fiber is directed towards the spiral ganglion neurons (green). The distance between the tip of the optical fiber and the spiral ganglion neurons was 200 µm. The optical fiber is sheathed in plastic tubing (PT) that passes through the metal bulla mount (blue) to secure the assembly to the bulla. The aABR threshold (bottom) shows an ∼30 dB elevation at high frequencies. (b) The microCT of a control cochlea shows that the optical fiber is inserted through a cochleostomy in the basal turn of the cochlea but is not pointed towards the spiral ganglion cells. Neither a behavioral nor electrophysiological response was observed in this animal. The distance between the tip of the optical fiber and the spiral ganglion neurons was 200 µm. The ABR threshold shows an ∼30 dB elevation at high frequencies. The difference in ABR thresholds below 10 kHz between the animals could be caused by variations in the surgical implantation or individual wound healing responses.

In five implanted cochleae (two that showed behavior and oABR responses, three without), a post-mortem microCT scan documented the orientation of the implanted optical fiber. Fig. 6a shows an example from a cat that had positive behavioral and electrophysiological responses, in which the optical fiber was oriented towards the spiral ganglion neurons (shown in green in the sketch). As measured from the microCT images, the distance from the tip of the optical fiber to the spiral ganglion neurons was about 200 µm. The oABR threshold for this animal was 5 µJ/pulse. This cat also demonstrated a behavioral response when the laser was turned on. In the other two stimulated cats, the distances from the tip of the optical fiber to the spiral ganglion neurons were 200 µm and 1600 µm. In the animals that did not show a behavioral or oABR response to laser stimulation (n = 3), the corresponding microCT scans showed that the optical beam path did not include spiral ganglion cells (an example is shown in fig. 6b). These cats did not show a behavioral response at the onset of laser stimulation, nor was there a measurable oABR, even at the highest laser energy available from the bench-top unit. Note, the post-operative aABR elevation is similar in the two cases in Fig. 6a and 6b (shown in the bottom panel of each figure).

### Histology

**Figure 7:**
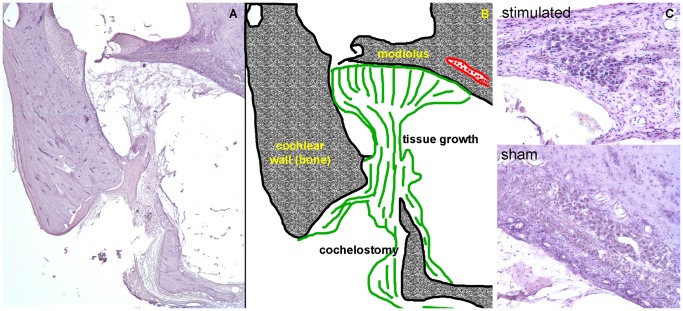
Histology reveals moderate tissue grown and no spiral ganglion neuron damage following chronic INS. (a) A cross section of the basal turn of the cat cochlea is shown with (b) the corresponding sketch. The optical fiber was inserted through the cochleostomy and directed toward the modiolus. Tissue (shown in green) formed around the optical fiber and remained mostly local. Thin strings of novel tissue fibers could be seen up to ∼1 mm from the cochleostomy toward the apical end of the cochlea. (c) Images of Rosenthal’s canal are shown for sham implanted and chronically stimulated cochlea. These sections show the location of the cochleae that were directly opposite the tip of the optical fiber. No sign of spiral ganglion neuron loss is apparent in the images.

Histology from sham implanted and stimulated animals did not show evidence of cochlear damage as a result of chronic laser stimulation. From the serial sections, the opening in the cochlear wall identified the cochleostomy. The opening was about 1.6 mm from the basal end of the cochlea. Post-implantation tissue growth occurred around the optical fiber. In all examples but one, the tissue was confined to the site of the implantation, as shown in figure 7. Tissue was found along the optical fiber, under the basilar membrane, and on the modiolar wall. Small “strings” of novel tissue were seen up to 1 mm away from the cochleostomy (the total length of the cat cochlear basilar membrane is ∼22 mm [35]). In one case, the entire scala tympani was filled with tissue. No loss of hair cells, pillar cells, or supporting cells was seen apical to the cochleostomy in stimulated cochleae. Spiral ganglion neurons were counted from serial sections, as described previously [34], [36]. Spiral ganglion neuron density was calculated by dividing the cross sectional area of Rosenthal’s canal by the number of neurons counted. The neuron density was similar in stimulated cochleae (1521±535 neurons/mm^2^; mean ± standard deviation) and in sham implanted cochleae (1441±381 neurons/mm^2^) (see also Fig. S1 and S2).

## Discussion

This manuscript presents behavioral data from a population of cats that were chronically implanted with an optical fiber for infrared neural stimulation (INS). Demonstrating that chronic, in vivo INS elicited a behavioral response is fundamental to developing new INS-based medical devices. The present study shows the potential of INS for applications in humans and adds to the existing literature examining the efficacy of infrared neural stimulation via acute electrophysiological measures or physical responses (e.g. compound action potentials from the 8^th^ nerve, muscle twitch, cardiac contraction) [3], [15], [37].

The cats demonstrated a behavioral response to an optical stimulus in the cochlea, as if searching for the source of a sound. Others have seen similar outcomes with electrically-stimulating cochlear implants [38]. The behavioral response to the optical stimulus reduced over the first 1–2 minutes after the stimulator was activated, indicating that an adaptation occurred to the laser stimuli. Adaptation is a mechanism by which specific neural responses decrease after prolonged exposure to a stimulus. Previous studies on human infants have shown an increase in behavioral response threshold over 3 successive presentations of an acoustic stimulus, indicating some adaptation to the test stimulus [39]. Studies on normal hearing adults have shown that the most significant adaptation to a continuous sinusoidal tone occurred during the first 1–2 minutes of presentation, depending on the stimulus frequency and sound level [40]. There are no known reports on the behavioral adaptation of animals that underwent cochlear electrical stimulation with which to make a direct comparison.

From the current experiments, it is not possible to conclude that the animals did hear a sound in response to cochlear INS. The data show that the animals exhibited a behavioral response that was correlated with the onset of the laser pulses. Also, the oABR data indicate that the brain received neural input from INS of the spiral ganglion neurons. However, further experiments are warranted to explicitly show that the animals perceive a sound, which can be determined in a behavioral training paradigm.

In this study, normal hearing animals were used, rather than chemically or genetically deaf animals, to assess the physiological state of cochlea and any damage that resulted from implantation and chronic stimulation. We observed a significant elevation of the post-operative aABR thresholds that was caused by the implantation. Similar results have been reported in the literature, demonstrating that a cochleostomy in the basal turn of the cochlea can elevate auditory thresholds, especially at high acoustic frequencies [41].

The post-stimulation aABR thresholds did not show a significant change from the post-operative aABR thresholds, indicating that there was little change due to the chronic laser irradiation in the cochlea (4–8 hours/day, up to 30 days). This is an important finding because it is known that there is a transient temperature rise associated with each laser pulse when the optical energy is absorbed by water [10]. With chronic INS at higher repetition rates (here 200 Hz), which will be required for cochlear implants, it is possible that the transient temperature rise summed into a steady state temperature increase that damaged the cochlea. Damaging temperature changes induced by chronic INS would be reflected in the aABR thresholds [42], [43]. There was no indication that thermal tissue damage occurred. Previous electrophysiology experiments, using INS at 200 Hz for up to 10 hours, did not show any signs of thermal damage to the cochlea [30]. A recent study demonstrated that thermal damage is only expected for radiant energies above 25 µJ/pulse at 250 Hz stimulation rate [44].

microCT imaging showed that the radiation beam must include the spiral ganglion neuron somata for successful INS. It is not sufficient to radiate the central projections of the spiral ganglion neurons. Previous results also showed that the optical path for INS must contain the neurons’ somata, rather than the axons [45]. While the stimulation of a smaller population of neurons is desirable and should benefit a cochlear implant patient, it may be a limitation of INS. Since INS targets a restricted volume of tissue in the beam path and the optical energy spreads very little, the stimulating source needs to be placed accurately to evoke a detectable response from the neurons [4], [11]–[13], [29]. Neurons must be in the optical path (generally co-aligned with the axis of the optical fiber) to be depolarized [45].

The typical wavelengths for INS are between 1840 and 2100 nm. Presence of fluids and tissue along the optical path will reduce the incident energy. Assuming that water is the sole absorber, these radiation wavelengths correspond to penetration depths of 100 to 1500 µm. In other words, the radiant energy at the tip of the optical fiber or at the radiation source decreases with each penetration length by about 67%. Therefore, INS-based neuroprostheses have to consider the distance, orientation, and absorbers between the radiation source and the target neurons. Minimizing the distance between the stimulating source and the neural tissue will increase the success of INS.

INS is based on a locally confined temperature change [10], [14], [46]. Local heating can also lead to a measurable pressure wave, which may directly stimulate the cochlea [47]. Several findings argue against such a mode of stimulation in cochlear INS. Pressure waves in the cochlea should vibrate the basilar membrane locally and deflect local hair cell stereocilia, leading to a neural depolarization that is acoustically driven. From the cochlear microCT scans shown in Figure 6, one would expect similar pressure wave generation and stereocilia deflection for both cases. However, the results in Fig. 6 show that neural depolarization occurs only in one of the two placements, in which the optical path included the spiral ganglion cell bodies. These results suggest that pressure wave generation is not the underlying factor for laser-evoked responses. Previous measurements of cochlear potentials during INS have shown a lack of cochlear microphonics, which arise from depolarizing currents through the sensory cochlear hair cells [15]. Neural responses from the cochlea could be evoked in chronically deaf gerbils using INS. Those animals had no cochlear hair cells and a largely reduced number of spiral ganglion neurons [18]. c-FOS staining after INS in gerbils revealed that neurons along the optical path were activated. Staining was found at one spot in the basal turn and another distinct spot one full turn higher [29].

Histology revealed no evidence for any cochlear damage caused by chronic INS in stimulated cochleae when compared to sham implanted cochleae. Structures, including spiral ganglion neurons at the same density, were present in both cochleae. According to the literature, outer hair cell loss typically presents within 1–2 days following cochlear damage. Spiral ganglion cell degeneration can begin ∼1–2 weeks following cochlear damage, typically following a loss of connected inner hair cells [48]–[50]. Results presented here are from animals that had an average of 4 weeks of chronic INS, sufficient time for cochlear cell degeneration to occur as a result of damage from INS. Chronically stimulated cochleae showed no evidence of thermal damage, as characterized by edema, rupture of the plasma membrane, cell shrinkage, nuclear fragmentation, vacuole formation, or coagulation [51].

In summary, this is the first known chronic application of INS. Behavioral responses validate that INS is successful in stimulating the cat auditory neurons. Long-term stimulation did not result in a change in the evoked responses, either INS- or acoustically-evoked. Histology shows no difference between chronically stimulated and sham implanted cochleae. The results suggest that INS-based neuroprostheses are promising tools to address human deficits. As optics technology progresses, it will determine whether an implantable cochlear array contains optical fibers or sources embedded in the array (e.g. VCSELs).

## Supporting Information

Figure S1 **Cochlear neuron density in sham vs. stimulated cochleae.** This graph shows the neuron densities in the basal turn of the cochlea for different animals. Sham implanted and stimulated animals have similar neuron densities in the cochlea. The open circles show the density calculated for each animal, while the filled circles show the mean ± standard deviation.
(TIF)

Figure S2 **Cochlear neuron density as a function of cochlear location.** Density values for one cochlea were obtained by stereological counting the neurons and subsequently dividing the counts by the cross sectional area of Rosenthal’s canal. Counting was performed by 2 individuals who were blinded to the other’s count. Density results are similar between the two individuals across the entire cochlea.
(TIF)

Movie S1 **Behavioral response seen after laser implant activation.**
(MP4)

Movie S2 **Behavioral response seen after laser implant activation.**
(MP4)
