# Supplementary figures and images for: Behavioral and Electrophysiological Responses Evoked by Chronic Infrared Neural Stimulation of the Cochlea
Source: PLoS One. 2013 Mar 7;8(3):e58189. doi: 10.1371/journal.pone.0058189 (PMC3591411; doi:10.1371/journal.pone.0058189)

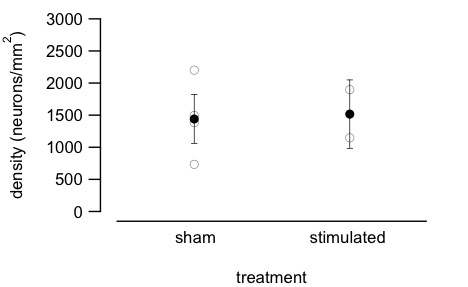

Supplement: Figure S1 — Cochlear neuron density in sham vs. stimulated cochleae. This graph shows the neuron densities in the basal turn of the cochlea for different animals. Sham implanted and stimulated animals have similar neuron densities in the cochlea. The open circles show the density calculated for each animal, while the filled circles show the mean ± standard deviation. (TIF) [file pone.0058189.s001.tif]

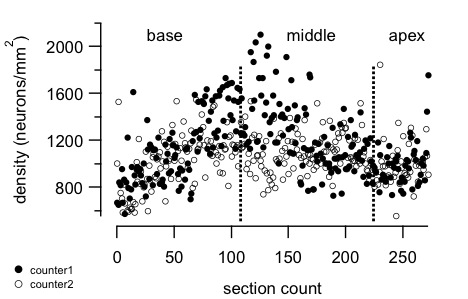

Supplement: Figure S2 — Cochlear neuron density as a function of cochlear location. Density values for one cochlea were obtained by stereological counting the neurons and subsequently dividing the counts by the cross sectional area of Rosenthal’s canal. Counting was performed by 2 individuals who were blinded to the other’s count. Density results are similar between the two individuals across the entire cochlea. (TIF) [file pone.0058189.s002.tif]
